# A low-cost, portable 32-channel EIT system with four rings based on AFE4300 for body composition analysis

**DOI:** 10.1016/j.ohx.2023.e00494

**Published:** 2023-11-18

**Authors:** Joel Escobar Fernández, Cristian Martínez López, Víctor Mosquera Leyton

**Affiliations:** a Universidad del Cauca, Street 5 No 4-70, Popayán, Colombia; b Universidad del Cauca, Electronic, Instrumentation, and Control Department, Street 5 No 4-70, Popayán, Colombia

**Keywords:** Electrical impedance tomography, Bioimpedance, Image reconstruction, Global impedance

## Abstract

A proposed low-cost, portable, 32-channel (4 rings of 8-channel) Electrical Impedance Tomography (EIT) system based on the AFE4300 analog front-end for body composition measurement. Each ring allows obtaining the conductivity distribution of 4 cross sections, 4 cm apart; to analyze the behavior of conductivity in a volume. The switching of the 4 rings and the current injection and voltage measurement patterns are done with three Texas Instruments 74HC4067 multiplexers, which are managed by an ESP32 board. The proposed system has an average signal-to-noise ratio of 74.71 dB and a frame rate of 50 fps. The sensitivity tests to impedance and volume changes consisted of introducing 4 tubes of different diameters (2 steel and 2 polyvinyl chloride) into a tank with saline solution; then conductivity distribution images were generated in 4 cross-sections of the tank, using the algorithms Gauss-Newton and Noser. Finally, the global impedance index (GI) is calculated to estimate the volume of each tube inside the tank. The results show that the proposed system is highly sensitive to impedance and volume changes, being a promising system for monitoring tissues, and fluids biological.

## Graphical abstract

**Figure:**
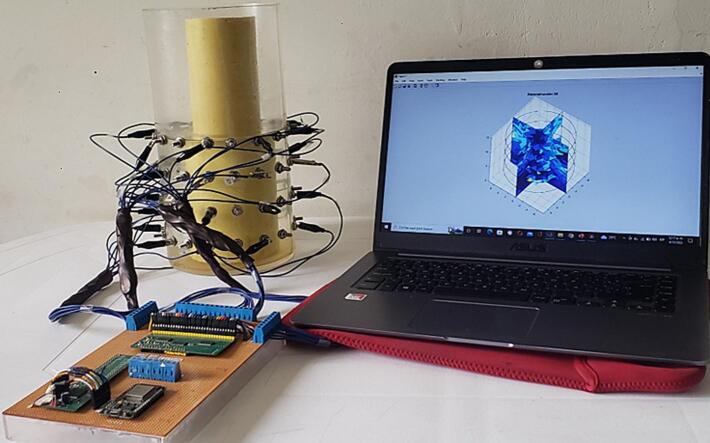

**Specifications table**

**Table:** 

Hardware name	*The name of the hardware that you have invented/customized*
Subject area	• Engineering and materials science
Hardware type	• Measuring physical properties and in-lab sensors • Field measurements and sensors
Closest commercial analog	*No commercial analog is available.*
Open source license	*CC BY 4.0*
Cost of hardware	*US$ 49.17*
Source file repository	*https://data.mendeley.com/drafts/zgdjg5b3cs**https://doi.org/10.17632/zgdjg5b3cs.1*

## 1 Hardware in context

Electrical impedance tomography (EIT) is a non-invasive method for visualizing the conductivity distribution of a cross-section (EIT-2D) or volume (EIT-3D) of interest. The technique injects low-intensity electrical currents and measures the resulting voltage changes through surface electrodes on the observed body [1], [2], [3]. Knowing the current and voltage measures, the inside conductivity distribution of the tissue can be calculated and then, employing an image reconstruction algorithm, to obtain the map conductivity [4]. EIT has significant potential in several applications, including medical imaging: for example, lung function [5], [6], [7], gastric emptying [8], [9], and detection of cancerous tissues [10], [11], among others.

EIT systems consist of 2 modules: i) the front end, which has a block for the alternating current signal, commutation blocks, and voltage readout, and ii) the back end, which manages the system and demodulates impedance [4], [12], [13], [14], [15]. Advancements in EIT systems aim to achieve high temporal and spatial resolution, which rely on the speed of signal processing (frequency of frames) and electrode number, respectively [4], [16], [17]. On the other hand, the most important characteristic is the signal-to-noise ratio (SNR) [17]; a high SNR indicates that the voltage measurement is more potent than the measurement noise, leading to a more precise EIT image reconstruction. Table 1 shows the features of EIT systems proposed for tissue biological monitoring.

**Table 1:** Features of recently published EIT systems.

Authors	SNR (dB)	Frequency of Frame (fps)	Number of electrodes	Management and switching devices
Yang et al. [18]	82.82	546	32	FPGA
Avery et al. [12]	77.5	100	16	Arduino Due/ADG714
Basak et al. [16]	–	–	8/16	Arduino UNO/CD74HC4067
Zamora et al. [13]	–	0.26	16	Arduino Mega/ADG1406
Rao et al. [19]	<76	10	20	FPGA
Muñoz et al. [20]	<59.97	100	8	AD5933/Arduino Mega
Brazey et al. [21]	<60.6	100	16	AD630ADZ/ADG506AKRZ
Wu et al. [17]	–	122	16	FPGA
Singh et al. [22]	70	70	16	Raspberry Pi/ CD4067BE
Li et al. [23]	81	100	16	FPGA
Mosquera et al. [24]	47.77	0.5	8	PIC16F886/AFE4300

The FPGA-based EIT systems implemented in [18], [17], [23] show a high performance (temporal resolution and SNR) compared to those that use a microcontroller, Arduino UNO, Mega, and so on [12], [13], [16], [22] (Table 1) as the main device; due to these devices allow the implementation of several blocks that compose an EIT system in a single component. Unlike those based on microcontrollers, which must ensure synergy between many digital and analog devices with which each block of the EIT systems is implemented. Another alternative to performing EIT includes commercial equipment such as signal generators [12], [21], being able to achieve high performance in terms of SNR and frame frequency, with the limitation of increasing implementation costs and decreasing system portability. Finally, [20] uses an impedance measuring device (AD5933) managed by an Arduino MEGA board, which presents a high frame frequency with a low SNR compared to the other proposed systems (Table 1).

Regarding spatial resolution, it is observed that for faster electrode management, researchers are leaning towards FPGA-based developments [18], [19], a device that allows for the agile setting of signal injection/measurement patterns and the number of electrodes; while other alternatives must adjust their designs and frame frequency to multiplexers [18], [16], [13], [21], [22], [24] or switches [12], [17], [19], which are limited by the switching speed or bandwidth (Table 1).

**Fig. 1:**
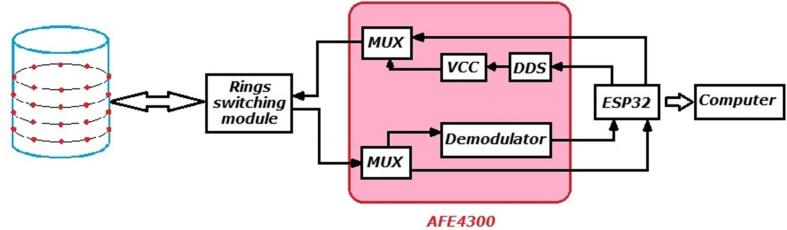
General scheme of 4R_EIT system.

In [24] shows an EIT system with eight electrodes based on AFE4300 integrated analog front-end for weight-scale and body composition measurement, which has a poorer performance (Table 1); but is low-cost, as it was the research aim. Considering that the AFE4300 device reduces the number of components for the development of an EIT system and its ease of management in terms of injection/measurement pattern programming [24], [25], this project presents the development of an EIT system of 32 electrodes, grouped into 4 rings (4R_EIT), together with the ESP32 for better performance. In Fig. 1, a modular diagram of the proposed EIT system is shown, in which the AFE4300 is responsible for generating, injecting, and measuring signals, and the ring switching module (4 rings of 8 electrodes) is composed of 3 TS3DV416 8x16 multiplexers. The ESP32 is responsible for the management of the AFE4300, the ring switching module, and transmitting measurements via Bluetooth or USB. The proposed 4R_EIT system independently manages the four 8-electrode rings, which generate images of the conductivity distribution in four different cross-sections of an object under study.

The SNR and frequency of frames will be calculated for the 4R_EIT system proposed for comparison with other EIT systems. The image reconstruction of conductivity distribution for each ring will be generated employing Gauss-Newton and Noser algorithms. Finally, the sensitivity of the 4R_EIT system to changes in the geometrical characteristics of objects under study will be evaluated using the global impedance (GI) index. The GI is a nondimensional index calculated for each EIT frame by summing the pixel values of the image reconstruction [26]. This index has a linear correlation with conductivity changes and shows promising results in the regional distribution of ventilation [27] and volume monitoring of the bladder [26].

## 2 Hardware description

The proposed 4R_EIT system allows for the estimation of the conductivity distribution in 4 cross-sections by using four rings of 8 electrodes located at different planes of the volume under study. The electrode arrays are managed by a ring switching module implemented with 3 TS3DV416 switches and an ESP32 board. The generation of the sinusoidal current signal and the measurement of voltage signals for each electrode ring is performed with the analog front-end AFE4300. The ESP32 card configures the AFE4300 registers to define the current signal frequency, injection, and measurement patterns. Finally, the EIT system communicates with a computer to store the measurements via Bluetooth or USB connection ( Fig. 1).

### 2.1 Front-end AFE4300

The AFE4300 is a mixed front-end with two modes of operation, one for weight measurement (Weight-Scale) and the other for body composition measurement (BCM). It incorporates a 16-bit, 860 SPS analog-to-digital converter (ADC).

**Fig. 2:**
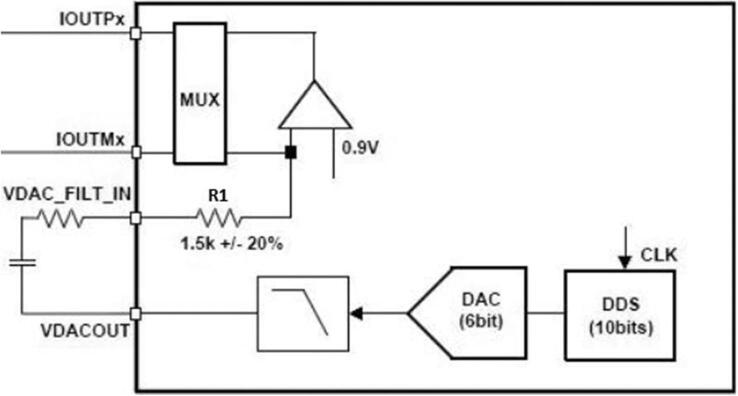
AFE4300 current generator block [25].

To measure BCM, 6-bit sequences at 1 MSPS are generated internally to produce a 1 Vpp sinusoidal signal; the signal frequency is programmed by a 1 MHz clock (CLK) inside the 10-bit direct digital synthesis (DDS) block and filtered by a low-pass filter at 150 kHz; subsequently. This signal is output through the VDACOUT pin, where a capacitor must be connected to remove the DC level and optionally a series resistor to reduce the amplitude. The signal enters the AFE4300 through the VDACFILTIN pin, and an operational amplifier generates a current signal (Voltage Current Controller (VCC) Source) that flows through two of the six ports IOUTx and the calibration pins of the device (RP0 and RP1) (Fig. 2).

The current generated by the AFE4300 is calculated by Eq. (1) and is smaller than the danger limits for the human body of 500 μAmp [28].

(1) $$i\left(t\right)=\frac{\mathrm{VDAC}}{R1}=\frac{1\;V_{\mathrm{pp}}}{1.5\;k\Omega \;\pm 20\%}\leq \frac{1\;V_{\mathrm{pp}}}{1.2\;k\Omega }=833\mu A_{\mathrm{pp}}$$

**Fig. 3:**
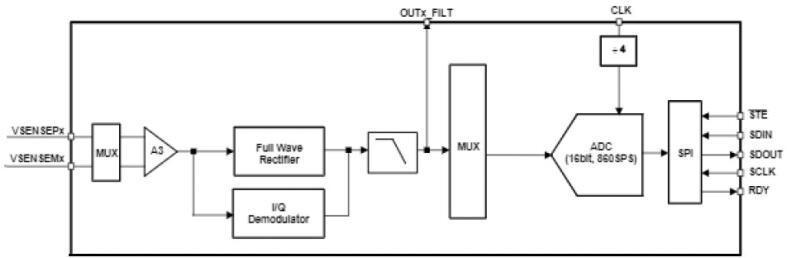
AFE4300 voltage measuring block [25].

The voltage difference generated by the injected current is measured using a pair of VSENSEX ports of the AFE4300. This measurement can be performed in two ways, by full-wave rectification (FWR) and demodulation in-phase and quadrature (Fig. 3). This project employs the FWR; for the measurement with this technique, the voltage signal is filtered to obtain a DC signal proportional to the impedance modulus (Eq. (2).

(2) DC=2T∫T2AZsinw0t+θdt=2AZπ=KZ

**Fig. 4:**
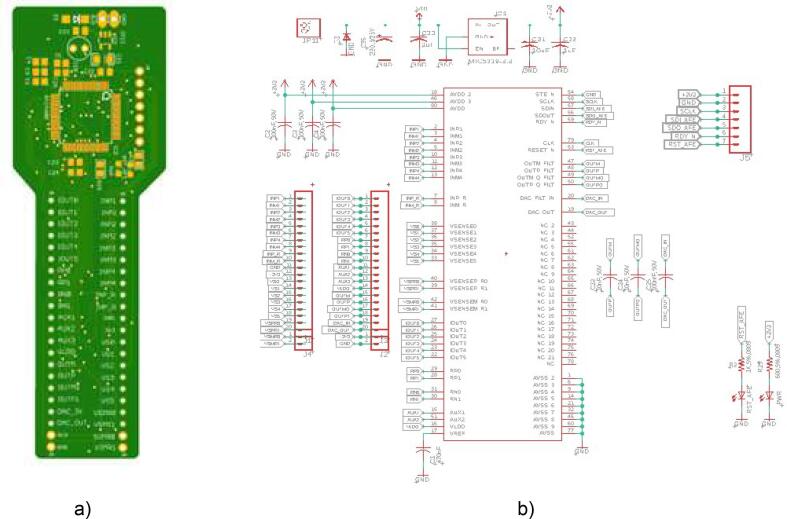
a) pcb afe4300 and b) scheme eagle of afe4300.

Where A, T, and $w_{0}$ are the current signal's magnitude, period, and frequency respectively and Z is the impedance under measurement. The PCB board of the AFE4300 is shown in Fig. 4.

### 2.2 ESP32

The ESP32 board has 24 Digital GPIO ports. This board operates with a voltage of 5 V in ideal conditions. In turn, has a 3.3 V power output for powering the AFE4300 and the TS3DV416 switch.

The ports used for communication with the AFE4300 are the following:

• Ports MISO (19) and MOSI (23): used to implement data transmission between the card and the AFE4300 using the SPI protocol.
• Port SCLK (18): a clock for SPI communication bus.
• Port CLK (15): 1 MHz clock for timing the AFE4300.
• Ports 3.3 V and GND: Power supply for the AFE4300 and the TS3DV416 card ring switching module.
• Port Reset (14): reset the board.
• Port RDY (12): informs when data is available.

Using the SPI communication protocol of the ESP32, full-duplex communication is established with the AFE4300. The register's configuration of AFE4300 is managed by the ESP32. These registers allow the operation in BCM mode, define the demodulation, and signal frequency definition, among others. The description of the more significative registers and the configuration values used for this project are presented in Table 1.

The registers configuration is in the folder ESP32_Files in the repository *https://data.mendeley.com/drafts/zgdjg5b3cs**.*

On the other hand, the ESP32 manages the rings switching module, implemented by three TS3DV416 switches, which allows obtaining the frames of each ring, Section 2.3 is explained this task.

### 2.3 Rings switching

**Fig. 5:**
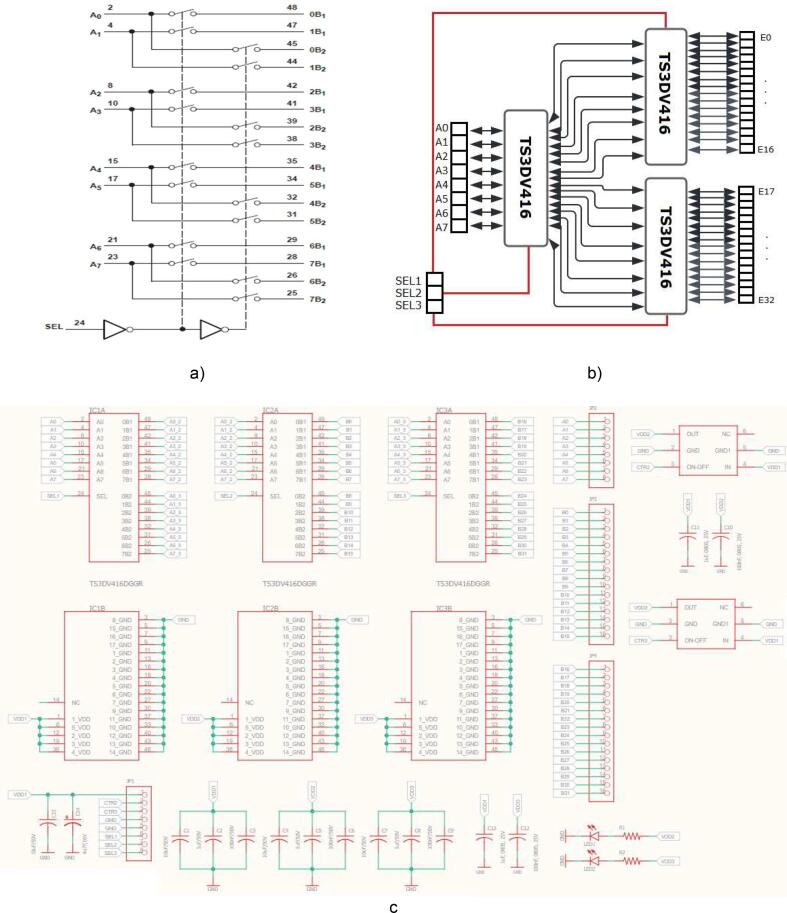
a) logic diagram of ts3dv416 [29], b) rings switching module connection, and c) scheme Eagle of rings switching module.

The ring’s switching module is designed in the EAGLE software according to the diagram in Fig. 5, this board has three TS3DV416 devices which make the multiplexing of 8 to 32 I/O, and each TS3DV416 has a digital control pin (SEL). The three select pins are enabled or disabled from the ESP32 for taking measurements from each electrode ring. The ***An*** inputs are connected to the AFE4300 current and voltage ports (IOUTx and VSENSEx).

Ports E0 to E7, E8 to E15, E16 to E23, and E24 to E31 are connected to the electrodes of rings 1, 2, 3, and 4, respectively. For each ring, the adjacent injection and measurement pattern is used. This pattern involves injecting current through adjacent electrodes and measuring the voltages across the other pairs of electrodes. For example, in a ring with 8 electrodes, the current is injected through electrodes E0-E1, and the voltages are measured across electrodes E2-E3, E3-E4, E4-E5, E5-E6, and E6-E7. Subsequently, the injection electrodes are rotated to E1-E2, and the voltages are measured across electrodes E3-E4, E4-E5, E5-E6, E6-E7, and E7-E0. The measurement process continues until the current is injected through electrodes E7-E0, and the voltages are measured across E1-E2, E2-E3, E3-E4, E4-E5, and E5-E6 [5], [20]. So, each ring generates 8X5 = 40 measures; this set of measurements is called a frame.

**Fig. 6:**
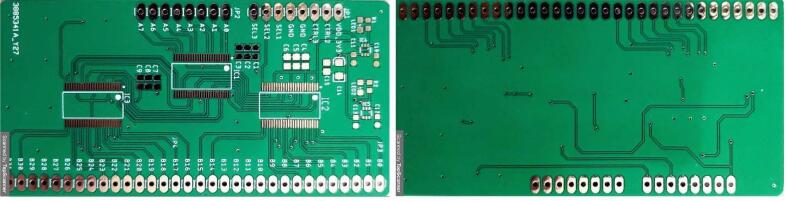
PCB board of rings switching module.

**Fig. 7:**
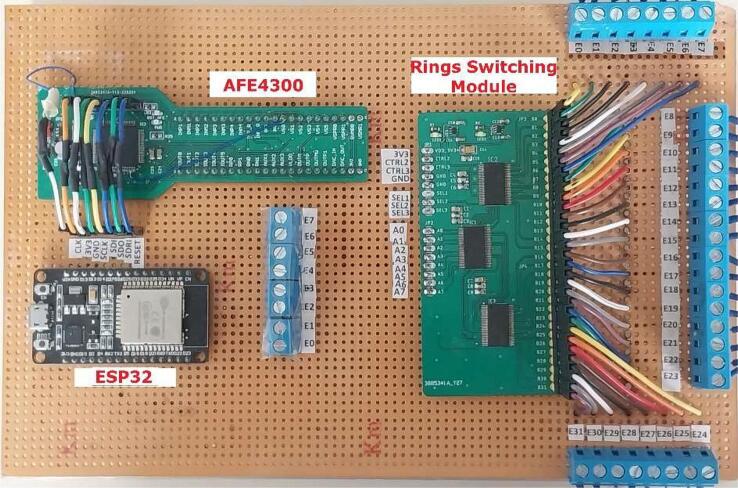
Assembled EIT system.

Fig. 6. shows the PCB rings switching module, the design file is in folder EIT_System_Design. Finally, the assembled 4R_EIT system is shown in Fig. 7.

## 3 Design files summary

Table 2 shows the figures that correspond to the design of the proposed 4R_EIT system.

**Table 2:** Description and configuration of AFE4300 registers.

REGISTER NAME	DESCRIPTION	VALUE
ADC CONTROL REGISTER1	The activation and operation mode of the ADC, also define the conversion speed, by default 128 SPS (Sample per Second) is used.	0x4140
DEVICE CONTROL1	Activate the body composition measurement mode.	0X6006
ISW MUX	Control for switches IOUTP, IOUTN, Rp, and RN	Variable
VSENSE MUX	Control for switches VSENSEP, VSENSEN, VSENSEP_R, AND VSENSEN_R	Variable
IQ MODE ENABLE 0x0C 0x0000	Enable IQ demodulation.	0x0000
ADC CONTROL REGISTER2	Defines the ADC output for BCM mode.	0x0063
BCM DAC FREQ	Define the frequency of the signal generated by the DDS	variable

The design files are in the 4R_EIT System folder of the repository *https://data.mendeley.com/drafts/zgdjg5b3cs**.*

• 4R_EIT_System_Design: This folder contains files corresponding to the design of the BIM system and their respective modules.
• PCB_Files: This folder contains the EAGLE design files of the PCB of 4R_EIT system.
• ESP32_Files: This folder contains the ESP32 sketch for operating the 4R_EIT system (files: afe_43000_current_bcm_mode.ino, afe_43000_current_inyection_matrix.ino, and TS3DV416_MUX_TEST.ino).
• Matlab_Script: This folder contains files corresponding to Matlab scripts used for serial communication (files serial_read_main.m), calculating the SNR (file SNR.m), calculating the global impedance and EIT image reconstruction (file Reconstruction3D_GI.m). In this folder, you will find several files containing measurements taken by the 4R_EIT system. These files include:
• 10_ms.txt, 20_ms.txt, 30_ms.txt, 40_ms.txt, and 50_ms.txt, which contain measurements obtained for the estimation test of the SNR (Section 6.1) at different measurement times.
• The files for both homogeneous measurements (Ring1_Reference.txt, Ring2_Reference.txt, Ring3_Reference.txt, Ring4_Reference.txt) and non-homogeneous measurements (Ring1_NoConductor.txt, Ring2_NoConductor.txt, Ring3_NoConductor.txt, Ring4_NoConductor.txt) for image reconstruction (Section 6.2).

## 4 Bill of materials summary

The list of materials used in the design of the 4R_EIT system is presented in the “Bill of Materials.xlsx”.

file in repository https://data.mendeley.com/drafts/zgdjg5b3cs.

## 5 Build instructions

**Fig. 8:**
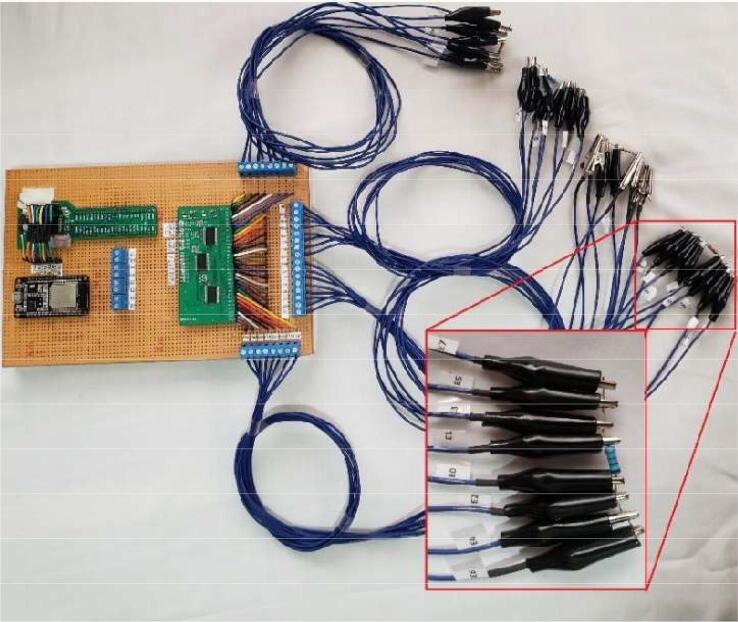
Connection to SNR estimation.
g. Finally, bias the 4R_EIT system with a 5-volt supply, which should be connected to the MIC5219 (IC1 of the AFE43000 PBC, Fig. 4a), supplying a 3.3-volt voltage to energize the modules of 4R_EIT.

For the proper functioning of the proposed 4R_EIT system (Fig. 7), the following building procedure should be considered:

a. Building of the PCBs of the AFE43000, and the ring switching module, according to the designs presented in Fig. 4a and Fig. 6.
b. Assemble the AFE4300 and the three TS3DV416 ICs on their respective PCBs. Additionally, solder the passive elements (resistors, capacitors, and LEDs) onto the PCBs according to the schematic diagrams in Fig. 4b and Fig. 5c.
c. Assemble the three modules, AFE4300, ESP32, and rings switching, on a single-sided universal PCB as indicated in Fig. 7. Use jumpers to connect the modules according to the schematic diagrams (Fig. 4b and Fig. 5C).
d. Assemble the three modules, AFE4300, ESP32, and ring switching, on a single-sided universal PCB as indicated in Fig. 7. Use jumpers to connect the modules according to the schematic diagrams of Fig. 4c and Fig. 5b. Finally, install four eight-pin terminal blocks on the universal PCB (Fig. 7) and connect them to the outputs of IC2 and IC3 of the ring switching module (Fig. 6).
e. Optionally, assemble a fifth terminal block to connect the first eight outputs of IC2 of the rings switching module (Fig. 6).
f. To carry out the tests with the 4R_EIT system, the following connections are made: i) For the SNR test, follow the connections indicated in Fig. 8. ii) Fig. 10 shows the connections for the reconstruction of EIT images. For this purpose, connect the first 8 outputs of IC2 of the ring switching module, to the 8 connectors of ring 1 and the other outputs of IC2 to the connectors of ring 2. The connectors of ring 3 should be connected to the first 8 outputs of IC3, and the remaining outputs of IC3 should be connected to the connectors of ring 4. The connection of each ring should be made considering the injection and measurement patterns depicted in Section 2.3.

## 6 Operation instructions

Once the 4R_EIT system is configured and energized, the measurements for the tests are obtained in the following manner:

### 6.1 Measurements to SNR

• Connect the 4R_EIT system as shown in Fig. 8.
• Load and execute the file “serial_read_main.m” located in *Matlab_Script* folder, to obtain the voltage measurements. These measurements are stored in the computer in a text file.
• Load file “SNR.m” located in *Matlab_Script* folder. In this script, modify the variable “A” and assign it the name of the file containing the voltage measurements obtained in the previous step and execute.

### 6.2 EIT image reconstruction and GI

• Connect the 4R_EIT system as shown in Fig. 10, following the instructions provided in Section 2.3 y 5f for the adjacent pattern.
• Load and execute the file “serial_read_main.m” located in *Matlab_Script* folder, to obtain the voltage measurements. For EIT image reconstruction experiments and GI calculation, homogeneous (Tank with saline solution) and non-homogeneous (Tank with saline solution and pipe) measurements should be taken and stored separately in the computer in a text file.
• Load file “Reconstruction3D_GI.m” located in the Matlab_Script folder and assign the homogeneous and non-homogeneous measurements obtained in the previous step to the variables 'Vh1,' 'Vh2,' 'Vh3,' and 'Vh4,' and 'Vi1,' 'Vi2,' 'Vi3,' and 'Vi4' respectively. The numerical indices of these variables (1, 2, 3, and 4) indicate the measurements of each ring.
• Finally, it will be possible to observe the conductivity distribution of the tank with saline solution in a 3D graph. In the Matlab command window, you can see the mean GI obtained.

## 7 Validation and characterization

### 7.1 Signal-to-noise ratio

**Fig. 9:**
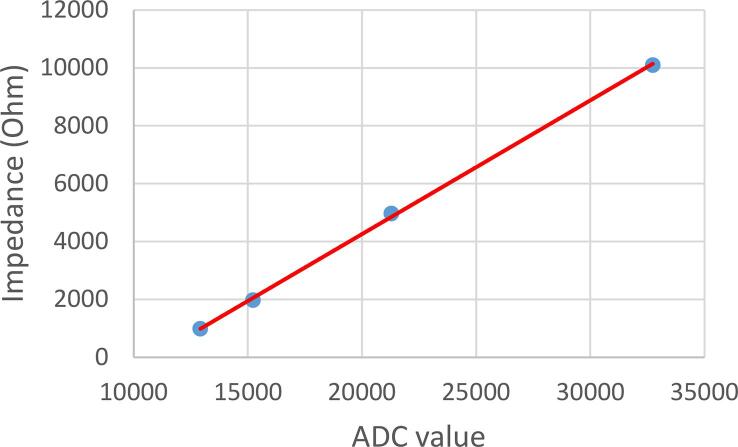
Linear regression of impedance measurement.

For the characterization of the proposed EIT system, measurement tests are performed on each ring for constant impedances of 997, 1980, 4980, and 10110 Ω (measures with multimeter FLUKE106); Fig. 8 presents the connections of electrodes of each ring for this experiment; which allow the same conductivity to be measured for each pair of electrodes. The magnitude of impedance measured by AFE4300 in FWR mode, employing a current signal of 50 kHz, shows a positive linear correlation (Fig. 9).

**Fig. 10:**
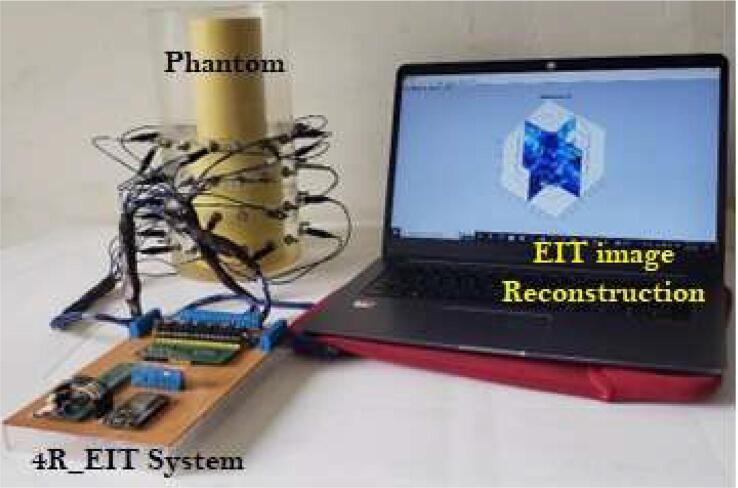
Assembly phantom experiments.

In addition, the voltage measurement time is varied (10, 20, 30, 40, and 50 ms) to determine the highest SNR (Eq. (3) that defines the frame rate.

(3) $$\mathrm{SNR}=20log\frac{mean\left(v_{i}\right)}{std\left(v_{i}\right)}$$

where $v_{i}$ are the measurements at each pair of electrodes. The SNR was calculated using 30 frames (1920 measurements) obtained from each impedance under study (Table 2).

According to Table 3, the highest SNR (74.71 dB) is obtained with a measurement time of 20 ms for a sample rate of 50 frames per second; this frame frequency will be used in the phantom experiments (Table 4).

**Table 3:** Desing files of 4R_EIT system.

**Design file name**	**File type**	**Open-source license**	**Location of the file**
General scheme of 4R_EIT system	Figure (PNG)	CC BY 4.0	Included in the article (Fig. 1)
PCB AFE4300	Figure (PNG)	CC BY 4.0	Included in the article (Fig. 4b)
Scheme Eagle of AFE4300	Figure (PNG)	CC BY 4.0	Included in the article (Fig. 4a)
Logic diagram of TS3DV416	Figure (PNG)	CC BY 4.0	Included in the article (Fig. 5a)
Rings switching module connection	Figure (PNG)	CC BY 4.0	Included in the article (Fig. 5b)
Scheme Eagle of rings switching module	Figure (PNG)	CC BY 4.0	Included in the article (Fig. 5c)
PCB board of rings switching module	Figure (PNG)	CC BY 4.0	Included in the article (Fig. 6)
Assembled EIT system.	Figure (PNG)	CC BY 4.0	Included in the article (Fig. 7)
Connection to SNR estimation	Figure (PNG)	CC BY 4.0	Included in the article (Fig. 8)
Assembly phantom experiments.	Figure (PNG)	CC BY 4.0	Included in the article (Fig. 9)

**Table 4:** SNR for different measurement times for 1kΩ.

**Measure time (ms)**	**Mean**	**High value**	**Low value**
**10**	58.13	62.96	55.35
**20**	**74.71**	**79.05**	**69.28**
**30**	59.28	63.26	54.70
**40**	59.51	63.66	56.14
**50**	57.94	63.56	54.61

### 7.2 EIT image reconstruction

To perform EIT image reconstruction tests, a phantom with four rings (7.5 cm in radius and 30 cm in height), two steel pipes (diameters of 0.5 and 1 in.), and two Polyvinyl Chloride (PVC) pipes (diameters of 1.5 and 3 in.), which ones are used to generating conductivity changes within the phantom (Fig. 10). An application of Matlab-EIDORS (Electrical Impedance Tomography and Diffuse Optical Tomography Reconstruction Software) was developed to carry out the EIT image reconstruction process. The reconstruction and regularization algorithms used in these tests were Gauss-Newton and Noser, respectively.

**Fig. 11:**
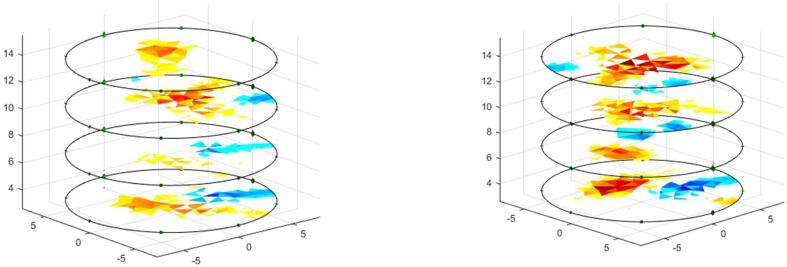
Image reconstruction for steel pipe of 0.5 in. diameter (left) and 1-inch diameter (right).

**Fig. 12:**
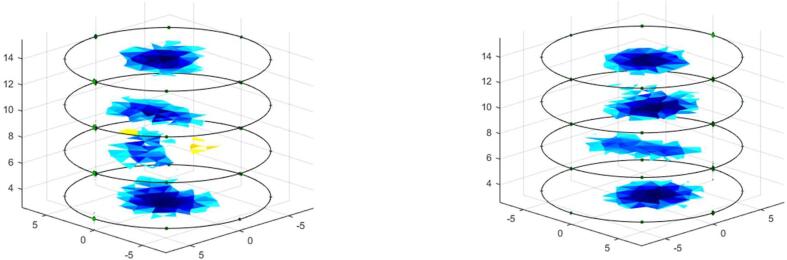
Image reconstruction for PVC pipe of 1.5-inch diameter (left) and 3 in. diameter (right).

The voltage measurements on the phantom with saline solution (2 gr/L) without pipes are called homogeneous measurements. One to one, the pipes are placed inside the phantom to obtain the non-homogeneous measurements. Both measurements are employed to determine the ability of the proposed system to detect variations in conductivity distribution and volume of the pipe. Fig. 11 and Fig. 12 show the reconstruction of conductivity distribution images for each phantom ring, for tests with steel and PVC pipes. Fig. 11 shows that the steel pipe presents a positive impedance change regarding the background (saline solution). Contrary, the PVC pipe gendered negative change in the conductivity distribution (Fig. 12).

**Fig. 13:**
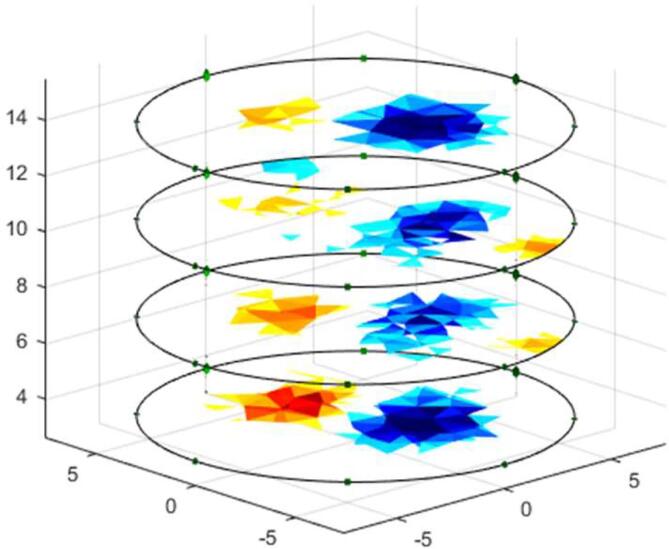
Image reconstruction for steel and PVC pipes simultaneously.

**Fig. 14:**
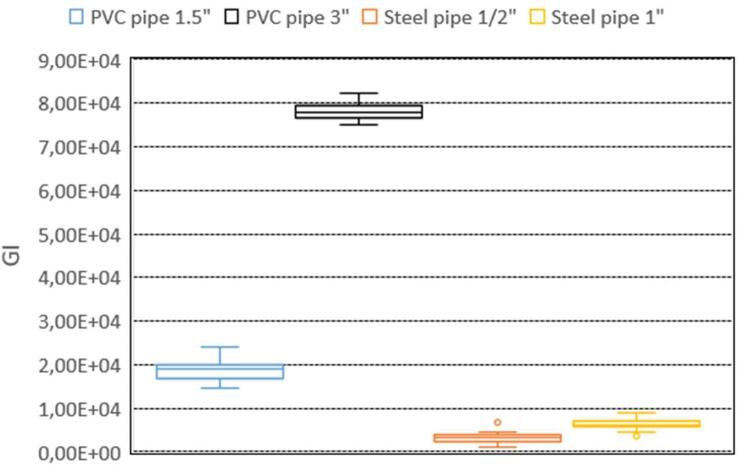
Volume estimation with GI.

Fig. 14 shows the volume estimation using the GI index; it can be seen that the 4R_EIT system allows differentiating the volume of the objects under study (steel and PVC pipes), making this system an alternative to analyze the size of objects and tissues.

Fig. 13 presents the reconstruction of EIT images when simultaneously introducing Steel (0.5-inch diameter) and PVC (1-inch diameter) pipes, showing that the 4R_EIT system allows for the detection of different impedance changes simultaneously.

To assess the 4R_EIT system response to changes in pipers’ size, the Global Impedance (GI) index is used, following the methodology described in [27], [30]. To conduct this experiment, each of the four pipelines used in the EIT image reconstruction tests is introduced individually, and 15 frames are captured for each pipe. The GI of the reconstructed images for each frame and ring is then calculated, and the average GI is obtained. Fig. 14 shows the volume estimation using the GI index. It can be noted that the 4R_EIT system allows differentiation of the volume of the objects under study (steel and PVC pipes).

The results obtained from the proposed 4R_EIT system demonstrate an SNR like that of FPGA-based EIT systems (Table 1) but at a lower cost. Additionally, a frame rate of 50 fps was achieved (Table 2), which enables the evaluation of conductivity changes in processes with high temporal variability, such as respiratory function and blood pressure, among others. Finally, the 4R_EIT system exhibits good sensitivity to volume changes when the GI index is used (Fig. 14). These results position the system as a promising alternative for biomedical applications aimed at monitoring impedance variation or volume changes. A study investigating the behavior of the 4R_EIT system for monitoring tissues and biological fluids at different ranges of frequencies will be conducted in the future.

## CRediT authorship contribution statement

**Joel Escobar Fernández:** Writing – original draft. **Cristian Martínez López:** . **Víctor Mosquera Leyton:** Conceptualization, Writing – review & editing.

## Declaration of Competing Interest

The authors declare that they have no known competing financial interests or personal relationships that could have appeared to influence the work reported in this paper.
