# Supplementary figures and images for: A low-cost, portable 32-channel EIT system with four rings based on AFE4300 for body composition analysis
Source: HardwareX. 2023 Nov 18;16:e00494. doi: 10.1016/j.ohx.2023.e00494 (PMC10767629; doi:10.1016/j.ohx.2023.e00494)

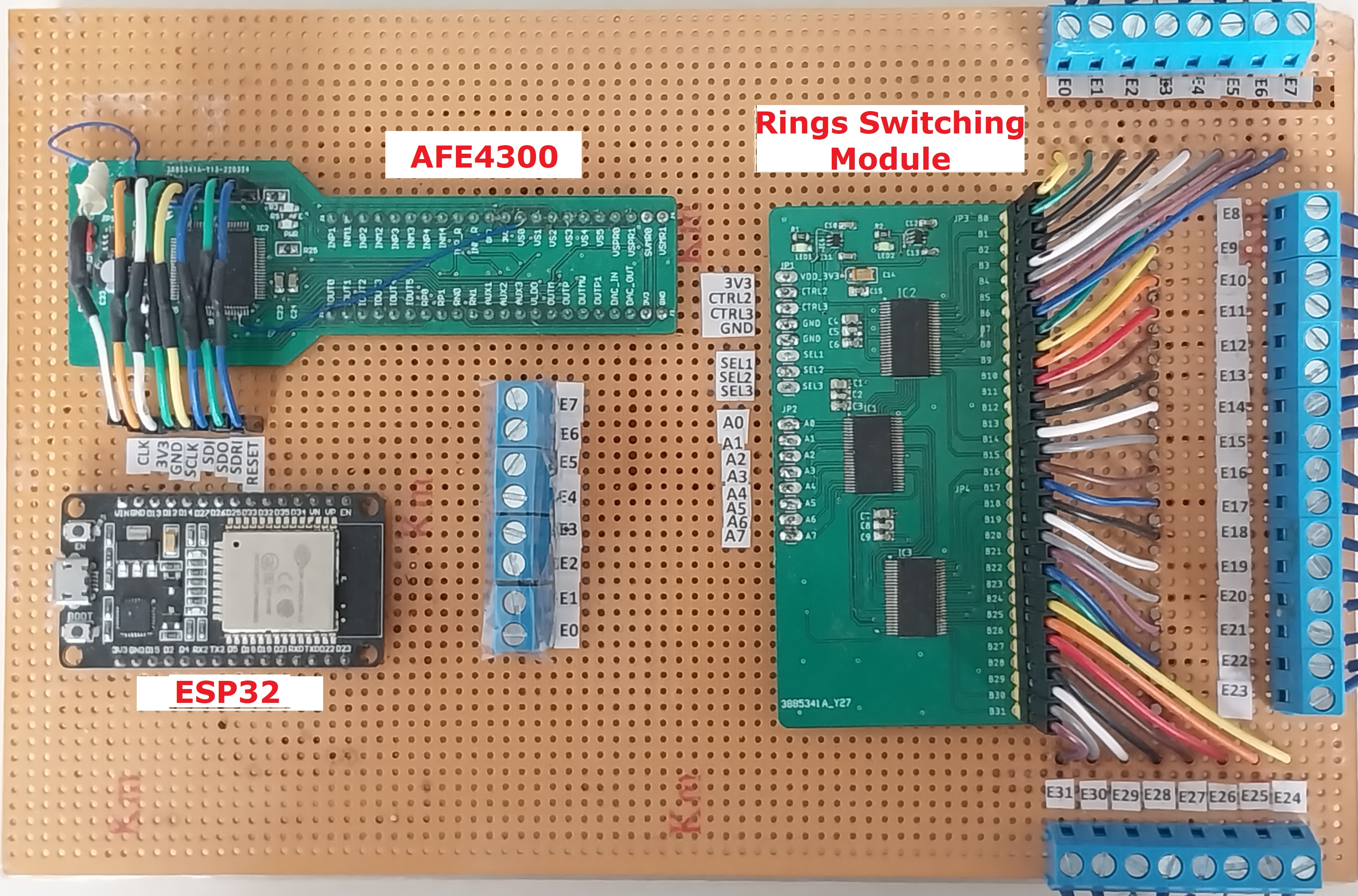

Supplement: Supplementary data 1 [file mmc1.zip › 4R_EIT_System_Design/Assembled EIT system.jpg]

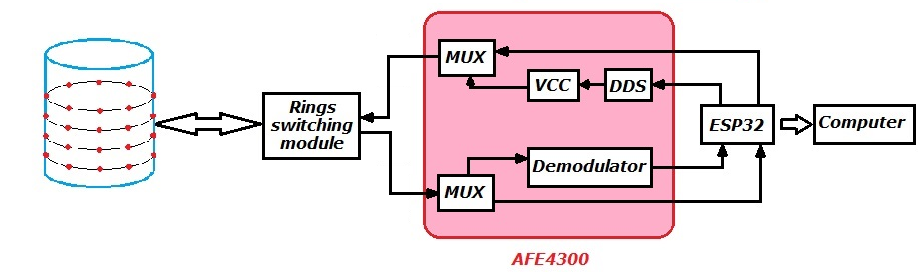

Supplement: Supplementary data 1 [file mmc1.zip › 4R_EIT_System_Design/General scheme of 4R_EIT system.png]

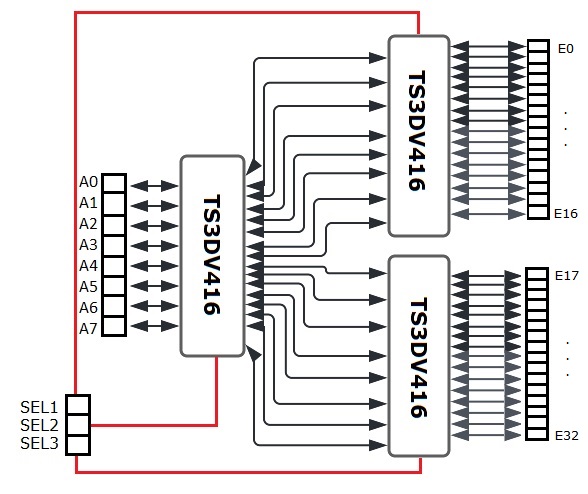

Supplement: Supplementary data 1 [file mmc1.zip › 4R_EIT_System_Design/Rings switching module connection.jpg]
